# Physiological effects of γ-aminobutyric acid application on cold tolerance in *Medicago ruthenica*

**DOI:** 10.3389/fpls.2022.958029

**Published:** 2022-11-07

**Authors:** Ying Li, Xiaojun Yu, Kaikai Ma

**Affiliations:** Key Laboratory of Grassland Ecosystem, Ministry of Education, Sino-U.S. Center for Grassland Ecosystem Sustainability, College of Grassland Science, Gansu Agricultural University, Lanzhou, China

**Keywords:** low temperature, GABA, antioxidation, photosynthesis, metabolism, leaf structure

## Abstract

Low temperatures in the seedling stage during early spring limit *Medicago ruthenica* germination and seedling growth. Elucidating the physiological mechanism of γ-aminobutyric acid (GABA)-regulated cold tolerance in *M. ruthenica* could provide a reference for alleviating the harmful effects of low temperatures on legumes in alpine meadows. The regulatory effects of GABA on *M. ruthenica* physiological parameters were explored by simulating the ground temperatures in the alpine meadow area of Tianzhu, China, in early May (2 h at 7°C; 6 h at 15°C; 4 h at 12°C; 2 h at 7°C; 10 h at 3°C). Our results showed that 15 mmol/l GABA was the optimal spray concentration to promote growth in the aboveground and belowground parts and increase the fresh and dry weights of seedlings. At this concentration, GABA enhanced the activities of catalase, peroxidase, superoxide dismutase, and ascorbate peroxidase; increased the osmotic balance; and inhibited the production of harmful substances in the cells under low-temperature conditions. GABA also regulated the tissue structure of leaves, increased the cell tense ratio, maintained photochemical activity, increased the amount of light energy to the photochemical reaction center, and improved the photosynthetic rate. Furthermore, exogenous GABA application increased the endogenous GABA content by promoting GABA synthesis in the early stages of low-temperature stress but mainly participated in low-temperature stress mitigation *via* GABA degradation in the late stages. Our results show that GABA can improve the cold tolerance of *M. ruthenica* by promoting endogenous GABA metabolism, protecting the membrane system, and improving the leaf structure.

## Introduction

*Medicago ruthenica* is a perennial herb with high crude protein content, good palatability, ecological width, and strong resistance to environmental and biological stressors (Yin et al., 2021). *M. ruthenica* is superior to *Medicago sativa* in terms of soil nutrient utilization and is more suitable for low-input cropping systems (Campbell et al., 1997). *M. ruthenica* mainly exists in Mongolia, Korea, and typical steppe and sandy steppe habitats in the low-temperature and cold temperate zones of northern China (Hao and Shi, 2006). Due to its good cold tolerance, *M. ruthenica* has the potential to be produced in the cold areas of China. Furthermore, it plays an important role in improving natural grassland, building artificial grassland, and improving the contradiction between grass and livestock (Xie et al., 2021). The alpine region lacks suitable legume species because of climate and environment problems; thus, *M. ruthenica* is an ideal species for natural grassland reseeding and artificial grassland planting in this region.

*M. ruthenica* is generally planted in early May each year in the alpine area of north China. Since the alpine grassland may suffer from prolonged low-temperature stress during sowing in early spring, it is preferable; moreover, it also affects plant yield and overwintering of *M. ruthenica*. In the Tianzhu Alpine Meadow in China, the average temperature in May has not exceeded 8°C in the last 30 years (Tong et al., 2021). Although *M. ruthenica* is cold-resistant, triggering antioxidant defenses when exposed to cold stress, plant defenses may be insufficient to mitigate extreme cold damage. Thus, additional substances may be needed to normalize plant growth at low temperatures. In recent years, the application of exogenous chemicals has become a common way to improve plant resistance. Exogenous chemical application has several ideal characteristics, such as economic viability, environmental safety, simple operation, strong applicability, and high effectiveness (Wang et al., 2021). Therefore, applying exogenous substances to improve the cold tolerance of *M. ruthenica* may be an effective approach for improving the utilization value of herbage.

γ-Aminobutyric acid (GABA) is a four-carbon, non-protein, free amino acid. Previous studies have focused on GABA as an inhibitory neurotransmitter in vertebrates to reduce the incidence of cardiovascular diseases (Xu et al., 2008). Later, GABA was used to regulate plant growth and development (Roberts, 2007; Beuve et al., 2004), especially under stress. GABA has been proven to effectively alleviate seed germination delay and growth retardation of plants under abiotic stress (Cheng et al., 2018), improving plant resistance. Most previous investigations on the regulatory effects of GABA have focused on salt stress (Kalhor et al., 2018), drought stress (Yong et al., 2017), and high-temperature stress (Liu et al., 2019). The alleviating effect of GABA on low-temperature stress has been observed in some fruits, vegetables, and crops, such as peach (*Prunus persica*) (Bustamante et al., 2016), wheat (*Triticum aestivum*) (Malekzadeh et al., 2012), and watermelon (*Citrullus lanatus*) (Palma et al., 2019); however, the effect of exogenous GABA application on *M. ruthenica* cold tolerance has not been reported.

In this study, we investigated the effects of the exogenous application of different GABA concentrations on the growth of *M. ruthenica* seedlings under low-temperature stress. The physiological mechanism of low-temperature regulation by GABA was evaluated by assessing changes in the antioxidant system, osmotic regulation system, photosynthetic fluorescence system, endogenous GABA metabolism, and leaf anatomical structure. Our findings will provide a reference for alleviating the harmful effects of low-temperature on legumes in alpine meadows.

## Materials and methods

### Plant material and growth conditions

*M. ruthenica* specimens were collected in Ningxian County, Gansu Province (35°33′N, 107°49′E, altitude 1,220 m), in 2015, planted in Huangyang Town in 2016, and harvested at the forage test station of Gansu Agricultural University, Huangyang Town, Wuwei City, Gansu Province (37°30′N, 103°15′E, altitude 1,660 m), in September 2018. *M. ruthenica* seeds were sown in containers (upper diameter 9.5 cm, lower diameter 5.0 cm, and height 10.5 cm) filled with vermiculite and distilled water, and all containers were randomly placed in the growth chambers (25/20°C (day/night), 70% relative humidity, and light for 12 h per day) for 7 days of germination. Plants were fertilized weekly with half-strength Hoagland’s nutrient solution (Hoagland and Arnon, 1950). Plants were transferred to light incubators after 1-month establishment in the growth chambers. The environmental conditions of light incubators were maintained at day/night temperatures of 25°C/20°C, 70% relative humidity, light for 12 h per day, and luminous flux density of 400 μ mol/m^2^·s. Plants were maintained in those conditions for 3 days before spraying GABA.

### Experimental design

The experiment comprised two parts: the GABA concentration screening test and the GABA regulation experiment. Six GABA spraying concentrations were used for the screening test, 0 (distilled water spraying, CK), 1, 5, 10, 15, and 20 mmol/l, respectively, with six repetitions per treatment. The low-temperature cycle was set to simulate the ground temperature at the forage experimental station of Gansu Agricultural University in early May (2 h at 7°C; 6 h at 15°C; 4 h at 12°C; 2 h at 7°C; 10 h at 3°C). The first three low-temperature cycling intervals were illuminated for 12 h per day, and the last two low-temperature intervals were set in darkness. The optimum GABA spraying concentration for promoting the growth of *M. ruthenica* was determined by measuring the growth indexes of plants. After the optimal GABA concentration was determined, four treatment groups were set up for the GABA regulation experiment: (1) leaves sprayed with distilled water + normal temperature (N); (2) leaves sprayed with 15 mmol/l GABA + normal temperature (NG); (3) leaves sprayed with distilled water + low-temperature stress (L); (4) leaves sprayed with 15 mmol/l GABA + low-temperature stress (LG). The normal-temperature culture conditions were 25°C (12 h per day)/20°C (12 h night); the low-temperature culture conditions were the same as those used in the GABA concentration screening test. Ten hydroponic boxes were set up for each treatment, six plastic bowls were placed in each hydroponic box, and each plastic bowl contained eight seedlings.

### Experimental methods

The leaves of *M. ruthenica* were sprayed with GABA on their abaxial and adaxial surfaces at 9:00 every morning. *M. ruthenica* seedlings were then put into the light incubator (25°C/20°C, 12 h/12 h, 12 h light/day) for 3 days and continuously sprayed for 3 days. On the fourth day, the seedlings were placed in an incubator that simulated the low temperatures of the Tianzhu Alpine meadow and exposed to light for 12 h/day. For the GABA concentration screening experiment, the seedlings were cultured for 15 days. The GABA regulation experiment seedlings were cultured for 35 days, and the physiological and photosynthetic indexes were measured at 0, 7, 14, 21, 28, and 35 days.

### Measurement of seedling growth index

On the day before low-temperature stress exposure, 30 seedlings were randomly selected from each treatment. The plant height (H1) of *M. ruthenica* was measured with a ruler and marked. At the end of the low-temperature stress exposure, the plant height (H2) was measured, and the plant height growth rate was calculated using the following formula. The root length and stem diameter were measured using a vernier caliper (mm); the leaf area was measured using a portable leaf area meter (Model: CI-203, CID Bio-Science, Inc., Camas, WA, USA). Five seedlings were randomly collected from each treatment and divided into aboveground and belowground parts. The fresh weight was measured using an analytical balance, and then the plant parts were dried in an oven at 105°C for 30 min, followed by 75°C until reaching a constant dry weight.


$$\text{Plant height growth rate}=({\text{H}}_{2}-{\text{H}}_{1})/{\text{H}}_{1}\times 100\%$$

### Measurement of relative electrical conductivity

Referring to the study of Zhang et al. (2020), leaves in the same position were collected and placed in a 10-ml centrifuge tube filled with ultrapure water for 15 h. The conductivity (EC) was measured using a conductance instrument (EC-TDS-NaCl-°C, Hanna Instruments, China). The tissue was inactivated by boiling for 25 min, and the conductivity (EC1) was measured again when the solution cooled to room temperature. The relative electrical conductivity (REC) was calculated as (EC/EC1) × 100.

### Measurement of oxidative damage

We referred to the methods of Li (2000) to measure the following indicators; malondialdehyde (MDA) content was determined by thiobarbituric acid colorimetry: 2 ml supernatant was mixed with 2 ml 2-thiobarbituric acid, incubated in a boiling water bath for 30 min, the absorbance measured at 450, 532, and 600 nm. The superoxide anion ( ${\text{O}}_{2}^{-}$ ) production rate was measured using the hydroxyl ammonia oxidation method: 0.5 ml supernatant was mixed with 0.5 ml phosphate-buffered saline (PBS, pH 7.8) and 1 ml of 10 mmol/l hydroxylamine hydrochloride in a centrifuge tube for 1 h at 25°C, then added to the reaction solution (1 ml of 17 mmol/l *p*-aminobenzene sulphonic acid and 1 ml of 7 mmol/l α-naphthylamine). The absorbance of the aqueous phase was determined at 530 nm. Hydrogen peroxide (H_2_O_2_) content was determined by potassium iodide–iodine spectrophotometry: 0.5 ml supernatant was added to 0.5 ml of 10 mmol/l potassium phosphate and 1 ml of 1 mol/l potassium iodide, and the absorbance was measured at 390 nm. An ultraviolet spectrophotometer (Q-6, Shanghai Metash Instruments, China) was used for all absorbance measurements.

Referring to the method of Li (2016), 2 mmol/l NBT was stained in 20 mmol/l PBS (pH 6.8) for 12 h, followed by ethanol decolorization and rinsing with deionized water, resulting in blue staining of ${\text{O}}_{2}^{-}$ . The leaves were stained with 0.1% (w/v) 3-diaminobenzidine (DAB; pH 3.8) for 24 h, decolorized with ethanol, and rinsed with deionized water, resulting in reddish-brown staining of H_2_O_2_.

### Measurement of antioxidant enzyme levels

We referred to the methods of Li (2000) to measure the following indicators. Catalase (CAT) activity was determined using the ultraviolet absorption method: 0.1 ml supernatant and 2.9 ml CAT reaction solution (100 ml of 0.15 mol/l PBS with pH 7.0 and 0.1546 ml of 30% H_2_O_2_) were mixed. Absorbance was recorded every 40 s at 240 nm. Peroxidase (POD) activity was determined using the guaiacol method: 40 μl supernatant and 3 ml POD reaction solution (50 ml of 0.2 mol/l PBS with pH 6.0, 28 μl guaiacol, and 19 μl of 30% H_2_O_2_) were mixed, and absorbance was recorded every 40 s at 470 nm. Superoxide dismutase (SOD) activity was determined *via* nitroblue tetrazole (NBT) reduction: 40 μl supernatant was mixed with 3 ml SOD reaction solution (162 ml of 14.5 mmol/l methionine, 26 ml of 3 mmol/l EDTA-Na_2_, 6 ml of 2.25 mmol/l nitroblue tetrazolium, and 6 ml of 60 μmol/l riboflavin). To assay ascorbate peroxidase (APX) activity, 0.1 ml of enzyme solution and 2.9 ml of APX reaction solution (2.60 ml of 0.05 mol/l PBS with pH 7.0 [containing 0.1 mmol/l EDTA-Na_2_], 0.15 ml of 5 mmol/l ascorbic acid, and 0.15 ml of 20 mmol/l H_2_O_2_) were mixed, and change in absorbance was measured at 470 nm after 40 s.

### Measurement of osmotic substance content

According to the method of Li (2000), the free proline (Pro), soluble sugar (SS), and soluble protein (SP) contents were determined. The free Pro content was determined by the acid ninhydrin colorimetric method: 0.5 g fresh sample was ground into a test tube with 5 ml of 3% sulfosalicylic acid and extracted for 10 min in boiling water. The supernatant (2 ml) and reaction solution (2 ml glacial acetic acid and 2 ml acidic ninhydrin) were put in boiling water for 30 min, 4 ml toluene was added to the mixture, and absorbance was measured at 520 nm. The SS content was determined by anthrone colorimetry: 0.1 g fresh sample was ground into a test tube with 5 ml distilled water and extracted for 30 min in boiling water. The supernatant (0.5 ml) was mixed with 1.5 ml distilled water and absorbance was measured at 630 nm. The SP content was determined by Coomassie Brilliant Blue G-250 staining: 1 ml supernatant was mixed with 5 ml Coomassie Brilliant Blue G-250 solution, and absorbance was measured at 595 nm.

### Measurement of photosynthetic parameters and photosynthetic pigments

The net photosynthetic rate (Pn), transpiration rate (Tr), stomatal conductance (Gs), and intercellular CO_2_ concentration (Ci) of leaves were measured from 9:00 to 11:30 using a Li-6400XT photosynthetic apparatus (LI-COR Biosciences, Lincoln, NE, USA). The determination light intensity was 400 μmol·m^−2^·s^−1^, the equipment’s leaf compartment was 3 cm^2^, and the CO_2_ concentration was set to 370 μmol·mol^-1^. The temperature setting of the leaf compartment was consistent with the temperature of seedlings in low- and normal-temperature incubators under the measured time. A fresh sample of the sixth leaf (0.1 g) was immersed in anhydrous ethanol and placed under dark conditions for 24 h. The absorbance of the supernatant was measured at wavelengths of 665, 649, and 470 nm. Then, we used the following formula to calculate the chlorophyll a, chlorophyll b, and carotene contents (Arnon, 1949).


Chlorophyll a concentration=13.95 A665−6.88 A649

Chlorophyll b concentration=24.96 A649−7.32 A665

Carotenoid concentration=(1000 A470−2.05 Ca−114.8 Cb)/245

Chlorophyll content=(pigment concentration×volume of extract)/fresh weight of sample

### Measurement of chlorophyll fluorescence coefficient

According to the method of Zhang (2019a), the following fluorescence parameters were determined. The sixth fully developed leaf was measured from 9:00 to 11:00 using a portable modulated chlorophyll fluorescence instrument (PAM-2100, WALZ, Germany). The initial fluorescence (F0) was determined after 20 min of dark acclimation, and the maximum fluorescence (Fm) was determined by irradiation saturation pulse (2,800.0 μmol·m^−2^·s^−1^). The steady-state fluorescence (Ft) under light adaptation was measured by switching on the endogenous photochemical light (600.0 μmol·m^−2^·s^−1^) for 5 min, and the maximum fluorescence (Fm′) under light adaptation was measured by switching on the saturation pulse (2,800.0 μmol·m^−2^·s^−1^) at 20-s intervals. Fluorescence parameters and leaf light energy distribution were determined according to the following formulas.


Potential photochemical efficiency (Fv/F0)=(Fm−F0)/F0

Maximal photochemical efficiency (Fv/Fm)=(Fm−F0)/Fm

Photochemical quantum yield (ФPSII)=(Fm′−Ft)/Fm′

Photochemical quenching coefficient (qP)=(Fm′−Ft)/(Fm′−F0)

Non-photochemical quenching coefficient (NPQ)=(Fm−Fm′)/Fm′

Photochemical electron transfer rate (ETR)=ФPSII×PAR×0.50×0.84

We determined the light energy distribution of the leaf, including the dissipation part of antenna [D = 1 − (Fm′ − F0′)/Fm′], the photochemical reaction part [P = qP × (Fm′ − F0′)/Fm′], the dissipation part of the reaction center [E = (1 − qP) × (Fm′ − F0′)/Fm′], and the excitation energy distribution imbalance between photosystem (PS)II and PSI: β/α − 1 = (Fm′ − F0)/(Fm′ − Fs)− 1.

### Measurement of endogenous GABA metabolism

The endogenous GABA content and glutamic acid decarboxylase (GAD) activity were determined using the Suzhou Coming test box method, and aminobutyrate transaminase (GABA-T) activity was determined *via* the Ruixin biological ELISA test box method (Yong et al., 2017).

### Measurement of the anatomical structure

The anatomical structure of plant leaves was determined according to the method of Zhang (2019b). Briefly, the sample was fixed in formalin–aceto–alcohol solution for more than 24 h. We used different gradients of alcohol for dehydration: 75% alcohol for 4 h, 85% alcohol for 2 h, 90% alcohol for 2 h, 95% alcohol for 1 h, anhydrous alcohol I for 30 min, anhydrous alcohol II for 30 min, alcohol benzene for 5–10 min, xylene I for 5–10 min, xylene II for 5–10 min, paraffin I for 1 h at 65°C, paraffin II for 1 h at 65°C, and paraffin III for 1 h at 65°C. The wax block was embedded and trimmed. Finally, the repaired wax block was cut into 4-μm pieces by a paraffin slicer, and the tissue was stained by ferro red–solid green staining and sealed. The leaf samples were observed on a light microscope (ECLIPSE Ci-L, Nikon, Japan). The thickness of leaf (LT), thickness of mesophyll (TM), thickness of upper epidermis (TUE), thickness of lower epidermis (TLE), thickness of palisade tissue (TPT), and thickness of sponge tissue (TST) were measured using Image-Pro Plus 6.0 analysis software. The palisade ratio, cell tense ratio, and spongy ratio were calculated using the following formulas.


Palisade ratio=TPT/TST

Cell tense ratio (CTR)=TPT/LT×100%

Spongy ratio (SR)=TST/LT×100%

### Data analysis and graphics

Microsoft Excel 2010 was used for data sorting. Values are presented as the mean ± standard error. IBM SPSS Statistics 26 software was used for variance analysis of the measured data, and Duncan’s new complex range method was used for multiple comparisons. The difference was considered statistically significant when *P*< 0.05. All images were created using Origin 2019.

## Results

### Effects of different GABA concentrations on the seedling growth index

**Figure 1:**
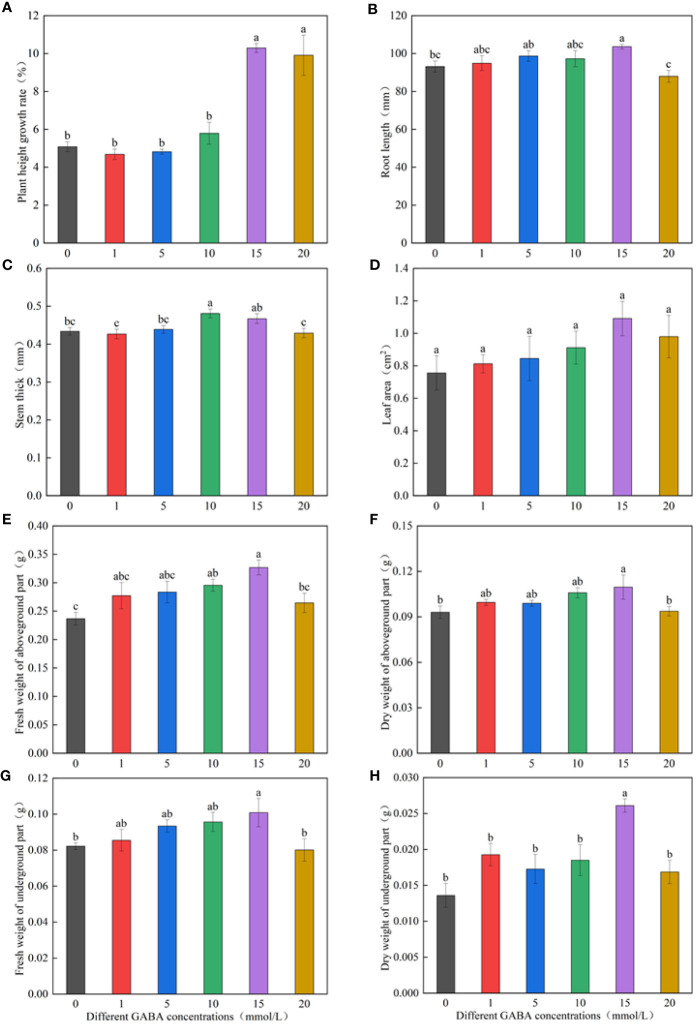
Effects of different GABA concentrations on seedling growth indexes. The growth temperature of seedlings is low-temperature in Figure **(A-H)**. Different lowercase letters indicate significant differences between treatments (*P*< 0.05).

**Figure 1** shows that the growth index first increased and then decreased with the increase in GABA concentration. The spraying concentration of 1–10 mmol/l GABA increased the growth indicators, such as the plant height growth rate, root length, stem diameters, leaf area, and dry weights; however, the difference was not significant compared with the increase obtained *via* the distilled water spraying treatment (control). The plant height growth rate in the 15- and 20-mmol/l GABA spraying treatments significantly increased by 102.39% and 94.91%, respectively, compared with the distilled water spraying treatment (**Figure 1A**). The GABA spray concentration of 15 mmol/l significantly promoted root elongation, increasing the root length by 11.28% compared with the control roots. The 20-mmol/l GABA spray concentration had an inhibitory effect on root length (**Figure 1B**). The stem diameters were largest in the 10-mmol/l GABA spraying treatment and the 15-mmol/l treatment, which were respectively 10.83% and 7.68% larger than the control (**Figure 1C**). The GABA spraying treatments did not significantly affect the leaf area (**Figure 1D**). The GABA spray concentration of 15 mmol/l increased the fresh and dry weights of seedlings by varying degrees (**Figures 1E–H**).

By measuring and calculating the height growth rate, root length, stem diameter, leaf area, and fresh and dry weights of *M. ruthenica* seedlings sprayed with different GABA concentrations, we concluded that the spray concentration of 15 mmol/l GABA was the optimal concentration to attenuate the effects of low-temperature stress on *M. ruthenica* growth.

### Effects of GABA on the phenotypic and oxidative damages of seedlings

**Figure 2:**
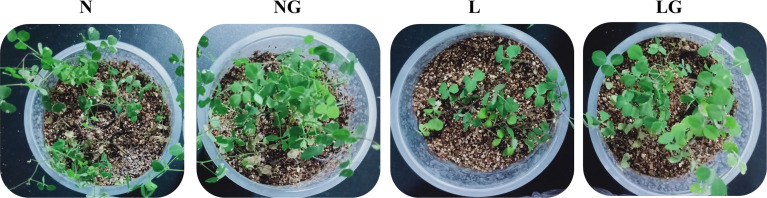
Effect of GABA spraying on the phenotypic characteristics of *M. ruthenica* seedlings under normal- and low-temperature treatments. N, leaves sprayed with distilled water + normal temperature (25°C/20°C); NG, leaves sprayed with 15 mmol/l GABA + normal temperature; L, leaves sprayed with distilled water + low-temperature stress (2 h at 7°C; 6 h at 15°C; 4 h at 12°C; 2 h at 7°C; 10 h at 3°C); LG, leaves sprayed with 15 mmol/l GABA + low-temperature stress.

**Figure 3:**
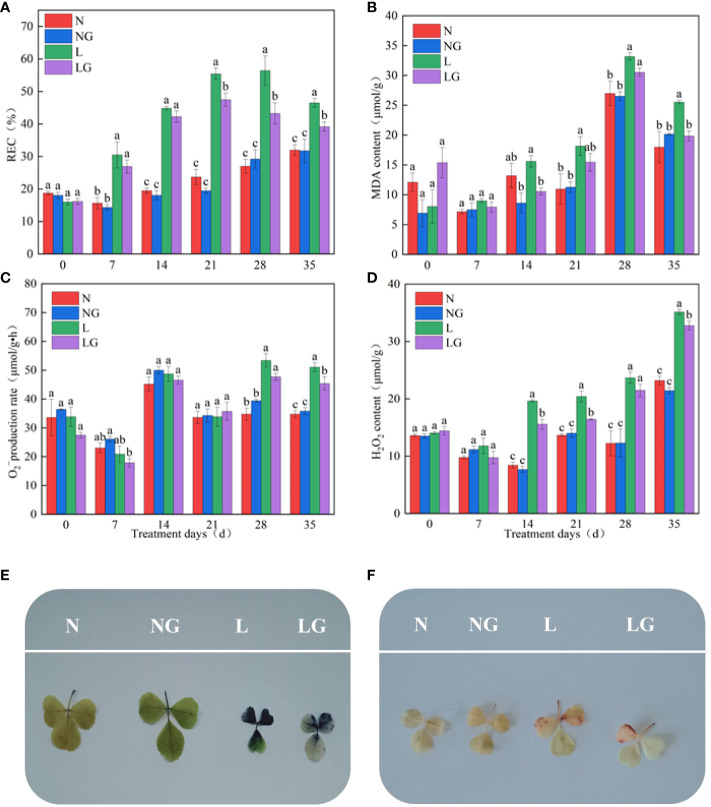
Effect of GABA spraying on oxidative damage parameters in *M. ruthenica* seedlings on different treatment days. **(A)** Relative electric conductivity (REC) content in leaves; **(B)** malondialdehyde (MDA) content in leaves; **(C)** superoxide anion ( ${\text{O}}_{2}^{-}$ ) generation rate in leaves; **(D)** hydrogen peroxide (H_2_O_2_) content in leaves; **(E)** visualization results of superoxide anion ( ${\text{O}}_{2}^{-}$ ); **(F)** visualization results of hydrogen peroxide (H_2_O_2_). N, the plant pretreated with distilled water and grown at normal temperature. NG, the plant pretreated with GABA and grown at normal temperature. L, the plant pretreated with distilled water and grown at low temperature. LG, the plant pretreated with GABA and grown at low temperature. Different lowercase letters indicate significant differences between N, NG, L, and LG on the same treatment day (*P*< 0.05).

Pre-spraying with GABA (LG) alleviated the effect of leaf wilting and poor growth caused by low temperatures (**Figure 2**). On days 21, 28, and 35 of the low-temperature treatment, the REC of LG significantly decreased by 16.81%, 30.44%, and 18.61%, respectively, compared with the L treatment (**Figure 3A**). On days 14 and 35 of low-temperature treatment, the MDA content of LG significantly decreased by 47.76% and 28.6%, respectively, compared with L (**Figure 3B**). On day 28, exogenous GABA alleviated the increase in the ${\text{O}}_{2}^{-}$ production. On day 35, the ${\text{O}}_{2}^{-}$ production rate of the LG was 12.43% lower than that of the L treatment (**Figure 3C**). The H_2_O_2_ content of LG significantly decreased by 26.08%, 24.23%, and 7.29% than L on days 14, 21, and 35, respectively (**Figure 3D**).

The NBT staining degree in the LG treatment was lighter than that in the L treatment, and there were fewer stained parts. Thus, GABA application alleviated the excessive ${\text{O}}_{2}^{-}$ accumulation at low temperatures (**Figure 3E**). The accumulation of H_2_O_2_ in seedlings was visualized by DAB staining. Fewer leaves were stained brown in LG than in L, indicating that GABA reduced the massive accumulation of H_2_O_2_ in seedlings under low-temperature stress (**Figure 3F**).

### Effects of GABA on antioxidant characteristics

**Figure 4:**
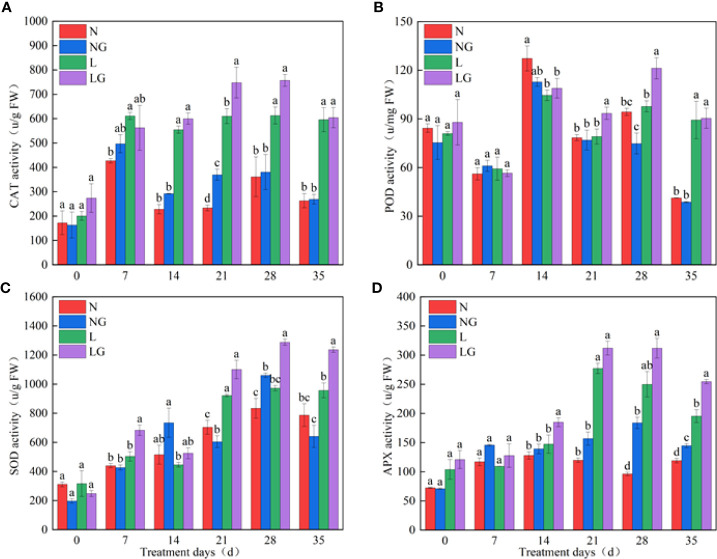
Effect of GABA spraying on antioxidant enzyme activity in *M. ruthenica* seedlings on different treatment days. **(A)** Catalase (CAT) activity in leaves; **(B)** peroxidase (POD) activity in leaves; **(C)** superoxide dismutase (SOD) activity in leaves; **(D)** ascorbate peroxidase (APX) activity in leaves. N, the plant pretreated with distilled water and grown at normal temperature. NG, the plant pretreated with GABA and grown at normal temperature. L, the plant pretreated with distilled water and grown at low temperature. LG, the plant pretreated with GABA and grown at low temperature. Different lowercase letters indicate significant differences between N, NG, L, and LG on the same treatment day (*P*< 0.05).

On day 21, GABA effectively enhanced CAT activity at normal temperatures (NG) and low temperatures (LG), and the CAT activity was highest in the LG treatment (**Figure 4A**). There was no significant difference in POD activity between N and NG. On days 21 and 28 of the low-temperature treatment, the POD activity of LG seedlings was significantly higher (18.91% and 24.08%, respectively) than that of L seedlings, and the enzyme activity showed a downward trend on the 35th day (**Figure 4B**). GABA spraying increased the SOD activity at normal and low temperatures. The activity of SOD was significantly higher (27.16%) in the NG treatment than in N. At low temperatures, GABA increased SOD enzyme activity by 35.81%, 32.45%, and 29.24% compared with that of L treatment on days 7, 28, and 35, respectively (**Figure 4C**). Compared with the N and L treatments, GABA spraying enhanced APX activity under both normal- and low-temperature conditions on days 28 and 35, and the activity showed a trend of LG > L > NG > N (**Figure 4D**).

### Effects of GABA on osmotic substance in seedlings

From the day 14 to day 35, the proline content of NG treatment was higher than that of N; however, the difference was not significant. At low temperature, pre-spraying GABA could increase proline content, which was significantly increased by 11.25% and 43.28% compared with L treatment on day 14 and day 35 (**Table 1**). The SS content in the N and NG treatments decreased from day 7 and then increased on day 35. The SS content in the L and LG treatments significantly increased from days 7 to 28 and decreased on day 35. The SS content in the LG treatment was highest on day 21, reaching 2.36 times that in the L treatment in the same period (**Table 1**). The SP content in LG was 18.85% and 31.08% higher than that in L on days 28 and 35, respectively (**Table 1**).

**Table 1:** Effects of GABA spraying on osmoprotectants in *M. ruthenica* seedlings on different treatment days.

Osmoprotectants	Treatments	Treatment days (d)					
		0	7	14	21	28	35
Pro (μg/g)	N	17.02 ± 1.02a	10.28 ± 0.26b	14.65 ± 1.52c	5.45 ± 1.62b	17.33 ± 2.94b	17.50 ± 1.23c
	NG	15.37 ± 3.04a	7.89 ± 1.29b	15.10 ± 0.61c	6.10 ± 2.68b	23.46 ± 4.13b	19.88 ± 4.07c
	L	12.88 ± 0.67a	145.52 ± 9.00a	272.27 ± 10.71b	53.13 ± 0.16a	192.55 ± 19.15a	133.85 ± 17.14b
	LG	15.54 ± 1.46a	286.92 ± 22.79a	302.89 ± 9.56a	63.26 ± 0.44a	226.57 ± 17.07a	191.78 ± 14.93a
SS (mg/g)	N	5.10 ± 0.22a	2.70 ± 0.27b	4.09 ± 0.43b	4.31 ± 0.75c	2.09 ± 0.14b	8.11 ± 0.65c
	NG	4.78 ± 0.61a	2.22 ± 0.41b	3.58 ± 0.26b	5.49 ± 0.49c	3.15 ± 0.80b	10.90 ± 0.66b
	L	4.34 ± 0.33a	7.06 ± 0.91a	24.96 ± 1.11a	16.91 ± 1.72b	33.00 ± 0.42a	17.45 ± 0.44a
	LG	6.27 ± 0.96a	7.46 ± 0.58a	23.43 ± 0.94a	39.98 ± 0.05a	35.02 ± 0.43a	17.86 ± 0.49a
SP (mg/g)	N	29.27 ± 3.76a	23.97 ± 0.88a	37.23 ± 2.88a	29.22 ± 0.6a	33.86 ± 1.99a	43.27 ± 3.01b
	NG	30.30 ± 1.85a	27.48 ± 0.57a	37.78 ± 1.26a	30.07 ± 1.44a	29.23 ± 0.94bc	50.51 ± 9.71ab
	L	31.04 ± 3.31a	25.68 ± 0.59ab	30.47 ± 1.04b	26.42 ± 1.32a	25.15 ± 0.45c	53.32 ± 10.02ab
	LG	35.83 ± 2.40a	19.72 ± 2.30b	29.78 ± 0.89b	28.18 ± 0.85a	29.89 ± 0.33ab	69.89 ± 4.01a

The contents of proline (Pro), soluble sugar (SS), and soluble protein (SP) in the four treatments of N, NG, L, and LG were measured on days 0, 7, 14, 21, 28, and 35. The first four lines represent the changes in Pro under different treatments, the middle four lines represent the changes in SS, and the last four lines represent the changes in SP. Under the same measurement indexes, different lowercase letters indicate the significant differences among N, NG, L, and LG on the same treatment days (P< 0.05).

### Effects of GABA on photosynthetic parameters and photosynthetic pigments of seedlings

**Figure 5:**
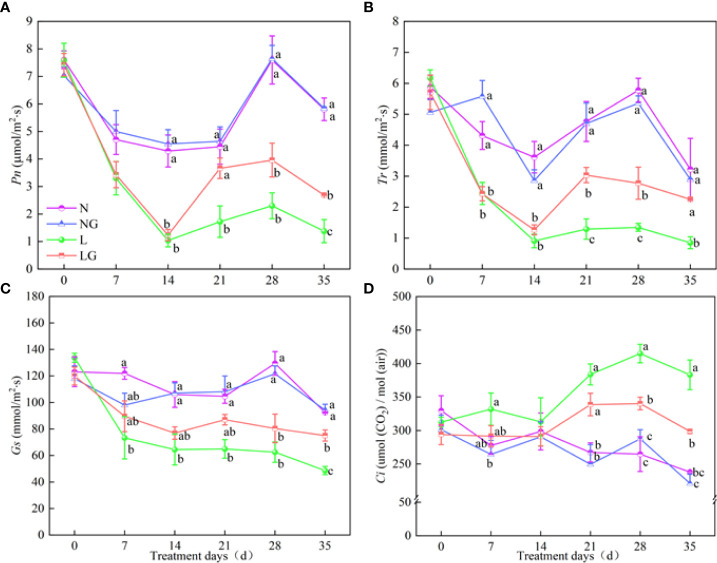
Effects of GABA spraying on photosynthetic parameters in *M. ruthenica* seedlings on different treatment days. **(A)** The net photosynthetic rate (Pn) in leaves; **(B)** transpiration rate (Tr) in leaves; **(C)** stomatal conductance (Gs) in leaves; **(D)** intercellular carbon dioxide concentration (Ci) in leaves. N, the plant pretreated with distilled water and grown at normal temperature. NG, the plant pretreated with GABA and grown at normal temperature. L, the plant pretreated with distilled water and grown at low temperature. LG, the plant pretreated with GABA and grown at low temperature. Different lowercase letters indicate significant differences between N, NG, L, and LG on the same treatment day (*P*< 0.05). Those not marked with letters under the same treatment day indicate that there is no significant difference between treatments.

The Pn of LG seedlings increased by 95.2% compared with L treatment seedlings on day 35 (**Figure 5A**). No significant differences in Tr, Gs, and Pn were observed between N and NG treatments. The Tr value in the LG treatment was 2.35, 2.06, and 2.65 times that in the L treatment on days 21, 28, and 35, respectively. The Tr of L and LG treatment decreased on days 0–28, and the Gs in the LG treatment was 54.39% higher than that in the L treatment on day 35 (**Figures 5B, C**). The Ci values in the L and LG treatments were higher than those in the N and NG treatments, and the Ci value was significantly lower (28.29%) in LG than in L on days 28 and 35 (**Figure 5D**).

**Figure 6:**
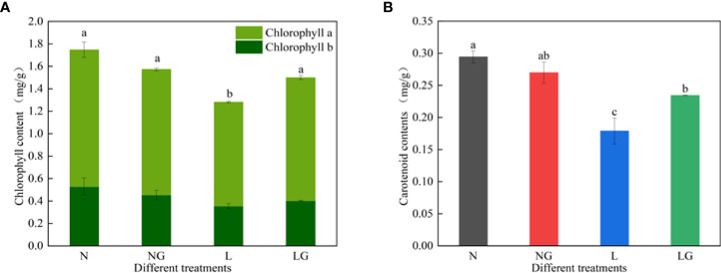
Effects of GABA spraying on photosynthetic pigment contents in *M. ruthenica* seedlings at normal and low temperatures. **(A)** Chlorophyll a and chlorophyll b content in leaves; **(B)** carotenoid content in leaves. N, the plant pretreated with distilled water and grown at normal temperature. NG, the plant pretreated with GABA and grown at normal temperature. L, the plant pretreated with distilled water and grown at low temperature. LG, the plant pretreated with GABA and grown at low temperature. Different lowercase letters indicate significant differences between treatments (*P*< 0.05).

The chlorophyll a, b and carotenoid contents decreased under low-temperature stress (**Figure 6**). Moreover, spraying GABA significantly increased the chlorophyll a and carotenoid contents at low temperatures but had no significant effect on the content of chlorophyll b. The chlorophyll a and carotenoid in LG were 18.48% and 31.07% higher than in L, respectively. The photosynthetic pigment contents were similar between N and NG treatments.

### Effects of GABA on chlorophyll fluorescence parameters

**Figure 7:**
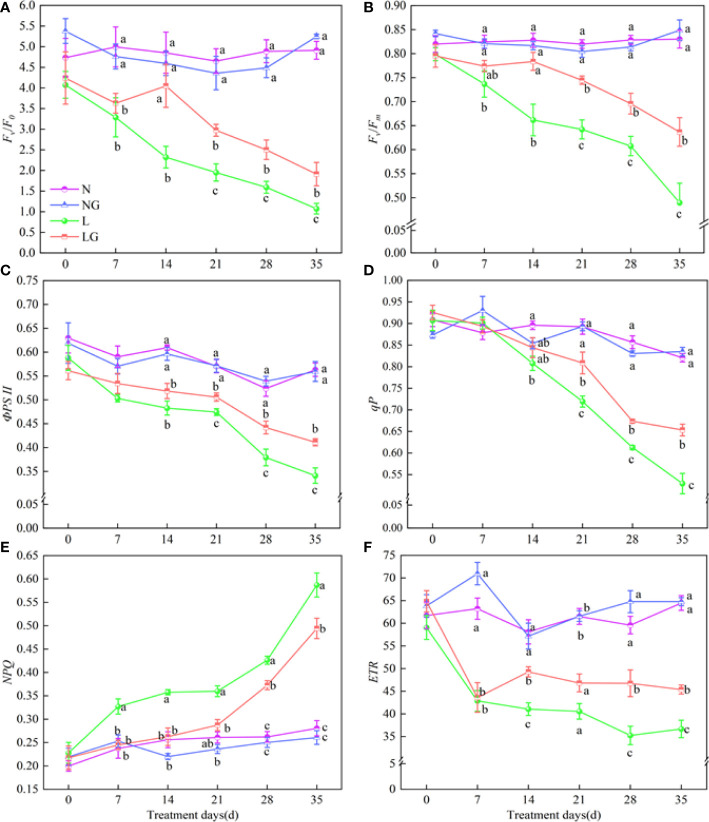
Effects of GABA spraying on the chlorophyll fluorescence parameters of seedlings on different treatment days. **(A)** Potential photochemical efficiency (Fv/F0) in leaves; **(B)** maximal photochemical efficiency (Fv/Fm) in leaves; **(C)** photochemical quantum yield (ФPSII) in leaves; **(D)** photochemical quenching coefficient (qP) in leaves; **(E)** non-photochemical quenching coefficient (NPQ) in leaves; **(F)** photochemical electron transfer rate (ETR) in leaves. N, the plant pretreated with distilled water and grown at normal temperature. NG, the plant pretreated with GABA and grown at normal temperature. L, the plant pretreated with distilled water and grown at low temperature. LG, the plant pretreated with GABA and grown at low temperature. Different lowercase letters indicate significant differences between N, NG, L, and LG on the same treatment day (*P*< 0.05). Those not marked with letters under the same treatment day indicate that there is no significant difference between treatments.

Fv/F0, Fv/Fm, ФPSII, qP, and ETR decreased and NPQ increased with increasing time in the low-temperature stress treatments (**Figure 7**). Compared with L, Fv/F0 in LG significantly increased by 52.46%–72.45%, and Fv/Fm in LG significantly increased by 15.94%–30.09% on days 14–35 (**Figures 7A, B**). From days 21 to 35, the ФPSII in the LG treatment increased by 6.62%–20.53%, and the qP significantly increased by 9.93%–23.43% compared with those in the L treatment (**Figures 7C, D**). NPQ was significantly higher in the low-temperature treatments. From day 7, the increase of NPQ in LG was much less than that in L. The NPQ was 18.79% lower in LG seedlings than in L seedlings on day 35 (**Figure 7E**). GABA also promoted increases in ETR, which were 20.05%, 32.69%, and 23.72% higher in LG than in L on days 14, 28, and 35, respectively (**Figure 7F**).

### Effects of GABA on absorbed light energy distribution in leaves

As the number of low-temperature stress days increased, the distribution of absorbed light energy to the dissipation part of the antenna (D) and photochemical reaction part (E) increased, whereas that to the dissipation part of the reaction center (P) continuously decreased (**Figure S1**). On day 35, the distribution of absorbed light energy to D and E was 41.85% and 36.82% lower in LG than in L, respectively, and the distribution to P was 27.08% higher in LG than in L, indicating that GABA spraying alleviated the energy dissipation at low temperatures (**Figures S1A-C**). β/α-1 continuously increased, showing a large energy imbalance between PSII and PSI at low temperatures. GABA alleviated this phenomenon, reducing β/α-1 in LG by 23.71%, 34.63%, 76.13%, 48.48%, and 27.54% compared with L on days 2, 14, 28, and 35, respectively (**Figure S1D**).

### Effects of GABA on the leaf anatomical structure

The leaves thickened irregularly at low temperatures. The LT in the L treatment was significantly higher than that in the N and NG treatments. Compared with L, the LG treatment significantly reduced the LT and mesophyll thickness at 14, 28, and 35 days (**Table 2**). The lower epidermis thickness of LG seedlings was significantly decreased by 27.51% and 23.16% on days 14 and 28, respectively, but GABA did not significantly affect the upper epidermis thickness (**Table 2**). Low temperatures also increased the TPT and TST. With continuous low-temperature stress, the arrangement of palisade tissue changed from compact to loose. On days 14, 28, and 35, the palisade tissue thickness was 29.65%, 10.59%, and 10.94% lower in LG than in L, respectively, and the sponge tissue thickness decreased by 35.19%, 28.95%, and 37.16%, respectively (**Figures S2**, **S3**, **Table 2**). The leaf palisade ratio and CTR gradually decreased as the low-temperature exposure time progressed, and the SR increased. GABA effectively alleviated the CTR decline caused by low temperature. Compared with the L treatment, the palisade ratio in LG increased significantly by 24.19% and the SR decreased by 13.45% after the low-temperature treatment (**Figure S2**, **Table 2**).

**Table 2:** Effects of GABA spraying on the anatomical structure of leaves of seedlings at low temperatures.

Treatment days (d)	Different treatments	LT (µm)	TM (µm)	TUE (µm)	TLE (µm)	TPT (µm)	TST (µm)	TPT/TST	CTR (%)	SR (%)
7	N	120.10 ± 1.60ab	92.77 ± 3.14a	18.98 ± 1.30a	13.98 ± 1.70b	51.73 ± 1.80a	40.83 ± 1.26a	1.27 ± 0.04a	43.15 ± 1.86a	34.01 ± 1.01a
	NG	115.01 ± 2.03b	84.93 ± 3.67ab	14.54 ± 1.09b	15.35 ± 0.21b	46.67 ± 0.72b	39.81 ± 2.80a	1.19 ± 0.07ab	40.60 ± 0.61a	34.58 ± 2.22a
	L	122.18 ± 2.71ab	80.46 ± 5.16b	18.68 ± 0.75a	20.09 ± 1.18a	41.16 ± 1.51c	41.95 ± 2.60a	1.00 ± 0.07c	33.79 ± 1.65b	34.33 ± 1.98a
	LG	124.20 ± 2.67a	88.11 ± 1.15ab	17.37 ± 0.78ab	17.33 ± 0.67ab	44.28 ± 0.99bc	42.82 ± 1.70a	1.04 ± 0.04bc	35.71 ± 0.99b	34.54 ± 1.60a
14	N	97.20 ± 1.33c	67.66 ± 2.30b	16.19 ± 0.70b	16.97 ± 1.13b	37.12 ± 0.96c	33.49 ± 2.33c	1.14 ± 0.10ab	38.26 ± 1.44a	34.43 ± 2.24ab
	NG	141.57 ± 0.78a	101.09 ± 2.95a	20.80 ± 0.96a	21.20 ± 0.92a	55.26 ± 1.69a	44.99 ± 2.28b	1.24 ± 0.07a	39.03 ± 1.17a	31.75 ± 1.44b
	L	138.73 ± 3.47a	104.44 ± 5.12a	18.31 ± 0.65ab	17.15 ± 0.74b	47.09 ± 1.67b	54.21 ± 2.87a	0.87 ± 0.03b	34.09 ± 1.80a	39.23 ± 2.59a
	LG	107.00 ± 3.25b	74.83 ± 3.51b	19.51 ± 0.83a	13.45 ± 0.68c	36.32 ± 1.54c	40.10 ± 3.72bc	0.95 ± 0.12b	34.10 ± 1.98a	37.26 ± 2.65ab
21	N	83.80 ± 1.29c	55.99 ± 1.87c	15.04 ± 0.87a	13.17 ± 1.44a	28.95 ± 2.12a	36.21 ± 2.83b	0.88 ± 0.10a	36.89 ± 2.20a	43.16 ± 3.12a
	NG	88.35 ± 1.91b	65.50 ± 0.70b	15.58 ± 2.18a	9.40 ± 1.00a	30.97 ± 1.10a	34.88 ± 0.55b	0.89 ± 0.04a	35.16 ± 1.64a	39.58 ± 1.29a
	L	112.71 ± 3.03a	82.60 ± 2.69a	16.26 ± 1.41a	13.96 ± 1.71a	32.01 ± 1.47a	50.30 ± 2.80a	0.64 ± 0.04b	28.43 ± 1.26b	44.64 ± 2.39a
	LG	110.55 ± 1.13a	78.48 ± 1.15a	17.68 ± 1.42a	14.07 ± 2.15a	29.26 ± 0.78a	51.57 ± 2.27a	0.57 ± 0.04b	26.49 ± 0.87b	46.69 ± 2.19a
28	N	81.27 ± 2.16d	52.12 ± 2.13c	11.78 ± 1.06b	13.61 ± 0.52ab	32.05 ± 0.77c	25.63 ± 2.40c	1.31 ± 0.15a	31.08 ± 0.41b	31.59 ± 2.99a
	NG	92.07 ± 3.34c	69.58 ± 4.64b	11.18 ± 0.65b	8.17 ± 0.40c	32.23 ± 0.62c	30.59 ± 1.16bc	1.06 ± 0.04ab	33.62 ± 0.60a	33.26 ± 0.82a
	L	125.41 ± 1.58a	87.90 ± 2.31a	22.05 ± 0.99a	15.05 ± 0.76a	42.17 ± 1.02a	44.50 ± 1.97a	0.95 ± 0.02b	33.62 ± 0.60b	35.44 ± 1.26a
	LG	113.91 ± 2.11b	73.99 ± 1.53b	20.40 ± 0.80a	12.22 ± 0.57b	38.13 ± 1.83b	34.51 ± 0.49b	1.11 ± 0.05 ab	32.99 ± 2.12b	30.35 ± 0.83a
35	N	79.54 ± 1.00c	65.21 ± 4.76bc	17.76 ± 0.92a	14.07 ± 1.09a	27.96 ± 1.64b	29.28 ± 0.61c	0.95 ± 0.05a	35.23 ± 2.33a	36.86 ± 1.19b
	NG	76.48 ± 2.98c	56.72 ± 4.01c	14.86 ± 0.66ab	10.57 ± 1.08a	26.37 ± 1.79b	29.33 ± 2.07c	0.91 ± 0.05a	34.35 ± 1.24a	38.20 ± 1.82b
	L	129.88 ± 0.72a	105.21 ± 1.12a	15.26 ± 1.32ab	13.91 ± 0.99a	36.41 ± 0.66a	59.15 ± 2.10a	0.62 ± 0.03c	28.05 ± 0.65b	45.55 ± 1.61a
	LG	107.33 ± 3.35b	70.83 ± 2.86b	14.10 ± 1.25b	14.19 ± 1.47a	32.82 ± 1.21a	43.03 ± 1.36b	0.77 ± 0.04b	30.79 ± 1.92ab	40.15 ± 1.11b

LT, leaf thickness; TM, thickness of mesophyll; TUE, thickness of upper epidermis; TLE, thickness of lower epidermis; TPT, thickness of palisade tissue, TST, thickness of sponge tissue; TPT/TST, palisade ratio; CTR, cell tense ratio, and SR, spongy ratio. Different lowercase letters indicate that there are significant differences among N, NG, L, and LG on the same treatment days and the same indexes (P< 0.05).

### Effects of GABA on endogenous GABA metabolism in seedlings

**Figure 8:**
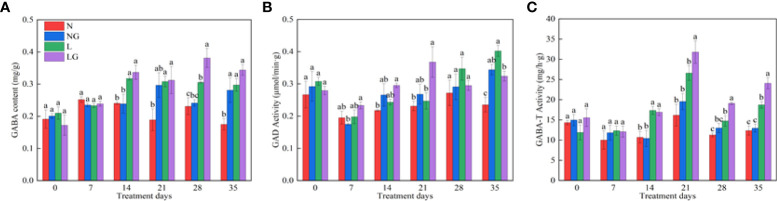
Effects of GABA spraying on the endogenous GABA metabolism of seedlings on different treatment days. **(A)** The endogenous γ-aminobutyric acid (GABA) content in leaves; **(B)** glutamic acid decarboxylase (GAD) activity in leaves; **(C)** aminobutyrate transaminase (GABA-T) activity in leaves. N, the plant pretreated with distilled water and grown at normal temperature. NG, the plant pretreated with GABA and grown at normal temperature. L, the plant pretreated with distilled water and grown at low-temperature. LG, the plant pretreated with GABA and grown at low-temperature.

As the number of treatment days increased, the endogenous GABA content of seedlings increased under low temperatures (**Figure 8**). The endogenous GABA content was highest in LG and was significantly higher (24.81%) than in L on day 28 (**Figure 8A**). GABA spraying also affected the seedling GAD. The GAD activity was highest in LG and was significantly higher (49.04%) than in L on day 21. After day 21, GAD activity began to decrease and was 24.16% lower in LG than in L on day 35 (**Figure 8B**). The GABA-T activity significantly increased in LG on the 14th day of low temperature and was 19.57%, 29.66%, and 28.66% higher than in L on days 21, 28, and 35 (**Figure 8C**).

## Discussion

Low-temperature stress can cause lipid membrane damage, physiological metabolism disorder, and cell structure destruction in plants (Liu et al., 2018). Higher conductivity and MDA content are markers of oxidative stress and reduced membrane stability and integrity. Our results showed that the electrical conductivity and MDA content increased as the low-temperature exposure time progressed, which reflects an increase in electrolyte leakage and cell membrane damage. GABA inhibits MDA formation during lipid oxidation (Deng et al., 2010), effectively protecting the membrane system (Li et al., 2016). GABA had a protective effect on the membrane in this study, delaying increases in relative conductivity and MDA content at low temperatures. Excessive reactive oxygen species (ROS) production can cause oxidative damage in plants under long-term stress (Mignolet-Spruyt et al., 2016), resulting in H_2_O_2_ and ${\text{O}}_{2}^{-}$ accumulation. Leaf staining showed that H_2_O_2_ and ${\text{O}}_{2}^{-}$ contents in leaves increased under low-temperature conditions. However, GABA attenuated the increases in H_2_O_2_ and ${\text{O}}_{2}^{-}$ , showing that GABA plays an important role in resisting oxidative damage. However, whether GABA can directly eliminate ROS is worth further discussion. Currently, no studies have confirmed that GABA directly removes ROS. The effect on ROS reduction observed in this study may be ascribed to the activation of some physiological mechanism by GABA to regulate the ROS removal process. We hypothesize that GABA may alleviate low-temperature stress by triggering antioxidant mechanisms. In this study, the activities of CAT, POD, SOD, and APX first increased and then decreased as the number of low-temperature days increased. The addition of GABA prevented reductions in antioxidant enzyme activities, enabling these enzymes to effectively remove ROS and protect cell membranes from damage. Our findings show that GABA enhances the cold tolerance of *M. ruthenica* by regulating the antioxidant system. The same conclusion was also reached in a study on wheat plants under salt stress (Li et al., 2016).

Plants usually rapidly synthesize and accumulate small molecular solutes (such as proline and soluble protein) to reduce water potential in plants and regulate osmotic pressure imbalance caused by environmental stress. In this study, the content of SS, Pro, and SP in seedlings at low temperature was higher than that at normal temperature. Exogenous GABA could be directly used as osmotic protectant or improve the osmotic adjustment ability of plants by increasing the content of soluble protein and proline to alleviate the damage caused by stress (Seifikalhor et al., 2019). In this study, the GABA treatment increased the free proline, soluble sugar, and soluble protein contents. This may be that exogenous GABA could improve the osmotic regulation ability of cells by increasing the content of osmotic substances, which can ensure the relatively normal growth of plants (Sikder et al., 2020). It may also be that GABA increases the contents of osmotic substances by upregulating enzymes related to the synthesis of these substances, such as Pro and trehalose, improving the growth of plant leaves under stress (Priya et al., 2019).

Photosynthesis is essential for plant growth and development. Stress will cause excessive light energy, resulting in light stress and excessive ROS production, damaging the photosynthetic mechanism, and hindering plant growth. The results reveal that the Gs and Tr decreased, indicating physiological activity and slower transpiration metabolism of plants at low temperature. In this study, Pn decreased and Ci increased at low temperature, which is related to the low temperature affecting the transport of CO_2_ to chloroplasts and inhibiting the normal progress of the Calvin–Benson–Bassham cycle. Low temperature may reduce the activity of enzymes involved in the Calvin–Benson–Bassham cycle, and there was no CO_2_ restriction (Tang, 2021). Thus, why could GABA improve the photosynthetic capacity of *M. ruthenica* at low temperatures? We speculate that adding GABA maintains the photosynthetic capacity of plants under stress by reducing plant water loss and increasing the transpiration rate under stress (Xu et al., 2021). GABA may also slow down the reduction rate of enzyme activity to maintain the photosynthesis, increase the excess light energy, and alleviate the reduction of the photosynthetic rate. In the growth diagram of this study, GABA alleviated the stunted growth of seedlings under low temperatures, which is related to the fact that GABA participates in the regulation of the photosynthetic system, protecting the photosynthetic mechanisms of seedlings under low temperatures and improving photosynthetic capacity. Chlorophyll content directly reflects the adaptation potential of plants to environmental changes (Wan et al., 2008). Carotenoid content can affect the absorption and utilization of light energy by plants (Fracheboud et al., 2004). In this study, both chlorophyll and carotenoid contents were reduced at low temperatures, indicating that stress may stimulate chlorophyll degradation, inhibit chlorophyll synthesis, or disorganize chloroplast tissue *via* photooxidation (Camejo et al., 2006). GABA may improve light utilization by increasing or decreasing photosynthetic pigment contents. Our results showed that the addition of GABA prevented decreases in chlorophyll a and carotenoid contents at low temperatures, indicating that GABA alleviates the rapid degradation of photosynthetic pigments. GABA may protect the pigments by reducing the excessive accumulation of chlorophyll a and chlorophyll precursors and maintaining the integrity of the chloroplast membrane structure (Xiang et al., 2016).

PSII is a major component of the photosynthetic electron transport chain and plays a key role in light energy conversion and electron transfer (Li et al., 2018). In this study, the chlorophyll fluorescence parameters related to the integrity of PSII changed at low temperatures, as Fv/F0, Fv/Fm, ФPSII, qP, and ETR decreased. These changes signify the damages to the PSII complex. GABA alleviated these effects, suggesting that GABA contributes to maintaining photosynthetic electron transfer, enhancing the activity of the photosynthetic reaction center, protecting PSII from damage, and increasing the absorption of light energy. In this study, a large amount of light energy was dissipated at low temperatures, and the energy used in the photochemical reaction center gradually decreased, indicating that low-temperature stress created an imbalance of energy in leaves, limiting the photochemical reaction capabilities of seedlings. GABA effectively attenuated the massive dissipation of plant light energy, promoted the distribution of light energy to the photochemical reaction center by redistributing leaf light energy, reduced the amount of heat energy dissipation, limited the excitation energy distribution imbalance between PSII and PSI, strengthened leaf photosynthesis, and improved the survival ability of plants under stress.

Leaf structure is an important index to evaluate the cold tolerance of plants. Under normal circumstances, the leaves of plants are thin, which is conducive to photosynthesis. Photosynthesis is weakened under stress; to carry out normal CO_2_ assimilation, the leaf structure changes. We found that the thickness of leaf significantly increased under low-temperature stress. This change may occur because small and thick leaves can adapt to stress by improving water storage and retention capacity. However, if the thickness of the leaf increases to a certain extent, the CO_2_ exchange capacity may be affected. Exogenous GABA application improved the leaf structure and prevented leaf thickening, not only enhancing the exchange and transportation capacity of CO_2_ and H_2_O but also protecting the leaf structure at low temperatures (Shi, 2016). The arrangement and the thickness of palisade tissue and sponge tissue reflect the adaptability of plants to the environment. We observed an increase in the thicknesses of palisade tissue and sponge tissue at low temperatures, particularly the sponge tissue, which is very unfavorable for leaf water retention. If the leaf structure of plants remains in this condition for an extended period, the photosynthesis and phenotypic characteristics of plants will be affected. The results of this study showed that adding GABA slowed down the overdevelopment of sponge tissue to maintain a reasonable proportion and improved the cell tense ratio, indicating that GABA can prevent the loose structure of plant leaves and weaken the negative effects of membrane lipid peroxidation and reduce photosynthesis at low temperatures. Considering that there are few studies on the effects of GABA on forage leaf structural characteristics, the results of this study will provide a reference for using GABA to improve leaf cell structures under stress.

The glutamic acid decarboxylation pathway is one of the important pathways of GABA metabolism. At low temperature, the synthesis of glutamine in plants is blocked, the synthesis of protein is reduced, and the transformation of glutamic acid to GABA in plants is increased under the action of glutamic acid decarboxylase (GAD) (Shi et al., 2007). Therefore, this study showed that the endogenous GABA content of the two groups of seedlings increased under low-temperature treatment, and the content of seedlings sprayed with GABA increased more significantly, indicating that spraying GABA on leaves could promote the transformation of glutamate into more GABA content. GABA accumulation and utilization result from the balanced regulation of its synthesis and degradation. When GABA accumulates, GABA-T plays an important role in the GABA degradation pathway. The substrate generated in this process participates in the tricarboxylic acid cycle of plants, which can enhance stress resistance (Ji, 2020). This study showed that the activity of GABA-T was weak and the GAD and GABA contents were high in the early stage of stress (0–14 days), indicating that the GABA synthesis pathway was active, and GABA played a positive feedback regulation role in endogenous GABA synthesis. During the middle stage of stress (21 days), the GAD activity and GABA-T activity in the LG treatment simultaneously increased, and the GABA content was also high, indicating that GABA accumulated to a certain extent at this time. In the later stage of stress (28–35 days), the GAD activity in LG decreased. The plant accelerates the degradation of GABA by enhancing the activity of GABA-T, resulting in the consumption of more GABA at low temperatures and the provision of higher ATP, promoting the tricarboxylic acid cycle, reducing the damage caused by low-temperature, and endowing plants with higher resistance (Fait et al., 2008; Podlešáková et al., 2019).

Therefore, we proposed that exogenous GABA alleviates the slim growth of *M. ruthenica* seedlings under low-temperature and enhances the cold tolerance, which is closely related to the antioxidant system, photosynthetic regulation system, light energy distribution, and leaf anatomical structure of plants. GABA could effectively remove ROS, reduce membrane system damage, improve cell osmotic regulation ability, and alleviate low-temperature injury. Moreover, the endogenous metabolism of GABA also plays a positive role in alleviating the low-temperature injury, accelerating the synthesis of GABA substances and providing substrates for the TCA cycle. Interestingly, in our study, GABA improved the growth of plants under low temperatures but did not significantly affect plants during the normal temperature treatment. This might be because GABA alleviates the low temperatures suffered by plants *via* the regulation of physiological mechanisms rather than providing only nitrogen sources.

## Conclusion

**Figure 9:**
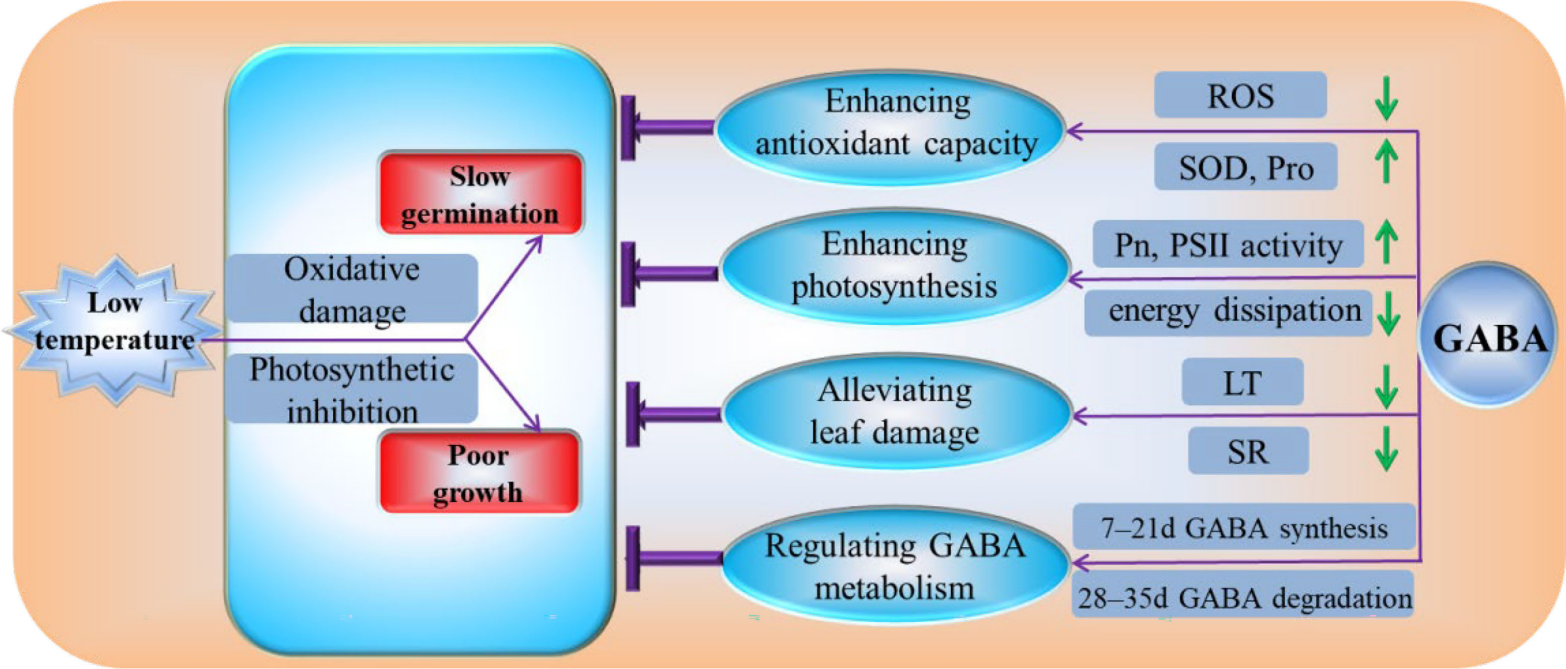
Physiological mechanism by which GABA regulates the cold tolerance of *M. ruthenica* under low-temperature conditions. ROS, reactive oxygen species; SOD, superoxide dismutase activity; Pro, the free proline; Pn, the net photosynthetic rate; PSII, photosystem II; LT, the thickness of leaf; SR, spongy ratio; GABA, γ-aminobutyric acid.

Our experimental results demonstrate that GABA can reduce the degree of membrane system damage by enhancing antioxidant enzyme activity, increasing the contents of osmotic substances, and reducing the relative conductivity, MDA content, and ROS generation at low temperatures. GABA increases the light energy used in the photosynthetic reaction center by increasing the photosynthetic rate and the activity of PSII in the photochemical reaction center. GABA protects leaf structures from cold damage by reducing the thickness of leaf, increasing the palisade ratio, and reducing the spongy ratio. GABA participates in low-temperature remission by regulating GABA synthesis in the early stress stage and GABA degradation in the later stress stage (**Figure 9**). Therefore, we believe that GABA is an effective chemical agent to alleviate low-temperature stress in plants. If GABA is applied to alpine meadow forages, it is expected to provoke a stress-priming response in plants before exposure to cold in early spring.

## Data availability statement

The original contributions presented in the study are included in the article/**Supplementary Material**. Further inquiries can be directed to the corresponding author.

## Author contributions

YL and XY conceived and designed the experiment. YL and KM conducted the experiment. YL analyzed the data and wrote the manuscript. XY revised the manuscript. All the authors read and approved the final manuscript.

## Funding

This research was funded by the Industrial Support Project of Universities in Gansu Province (No. 2022CYZC-50).

## Acknowledgments

The authors would like to thank NES for the English language review.

## Conflict of interest

The authors declare that the research was conducted in the absence of any commercial or financial relationships that could be construed as a potential conflict of interest.

## Publisher’s note

All claims expressed in this article are solely those of the authors and do not necessarily represent those of their affiliated organizations, or those of the publisher, the editors and the reviewers. Any product that may be evaluated in this article, or claim that may be made by its manufacturer, is not guaranteed or endorsed by the publisher.
