# Supplementary material for: Physiological effects of γ-aminobutyric acid application on cold tolerance in Medicago ruthenica
Source: Front Plant Sci. 2022 Nov 7;13:958029. doi: 10.3389/fpls.2022.958029 (PMC9676939; doi:10.3389/fpls.2022.958029)
Supplement: Supplementary file 1 [file DataSheet_1.pdf]

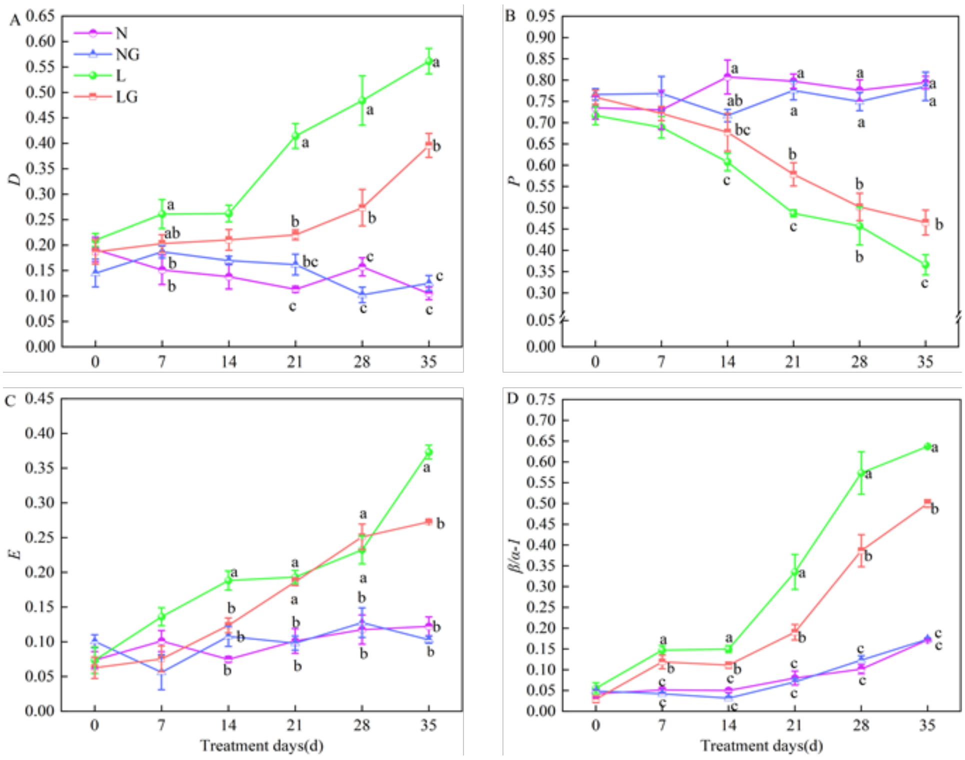


Fig. S1 Effects of GABA spraying on the distribution of light energy absorbed by seedling leaves on different treatment days. Fig. 8-A depicts the light energy in the dissipation part of antenna (D) in leaves; Fig. 8-B depicts the light energy in the dissipation part of reaction center (P) in leaves; Fig. 8-C depicts the light energy in the photochemical reaction part (E) in leaves; Fig. 8-D depicts the excitation energy distribution imbalance between photosystem (PS)II and PSI (β/α-1) in leaves. N, the plant pretreated with distilled water and grown at normal temperature. NG, the plant pretreated with GABA and grown at normal temperature. L, the plant pretreated with distilled water and grown at low-temperature. LG, the plant pretreated with GABA and grown at low-temperature. Different lowercase letters indicate significant differences between N, NG, L, and LG on the same treatment day ($P < 0.05$). Those not marked with letters under the same treatment day indicate that there is no significant difference between treatments.

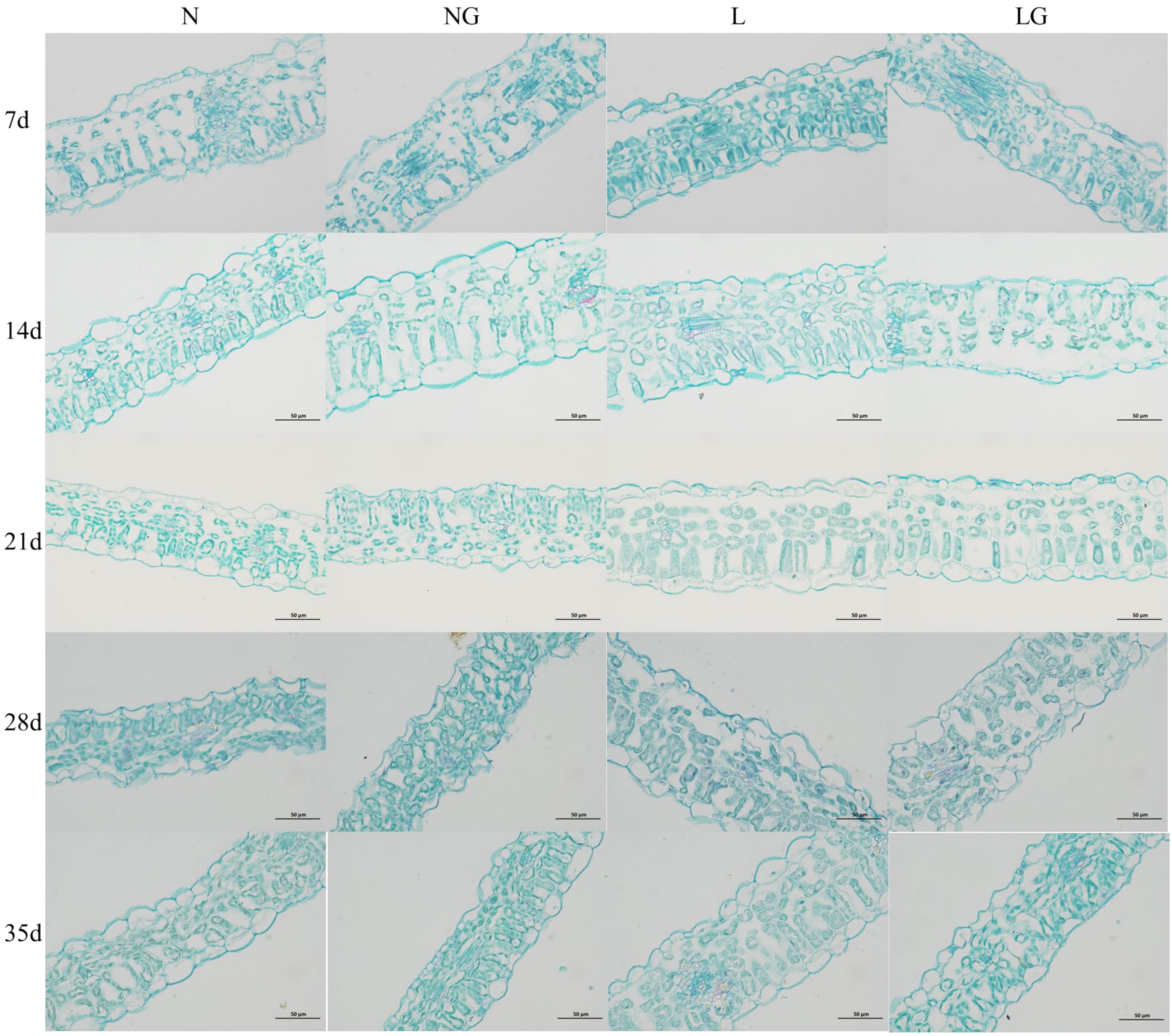


Fig. S2 Effects of GABA spraying on the anatomical structures of *M. ruthenica* seedling leaves on different treatment days. N, the plant pretreated with distilled water and grown at normal temperature. NG, the plant pretreated with GABA and grown at normal temperature. L, the plant pretreated with distilled water and grown at low-temperature. LG, the plant pretreated with GABA and grown at low-temperature.

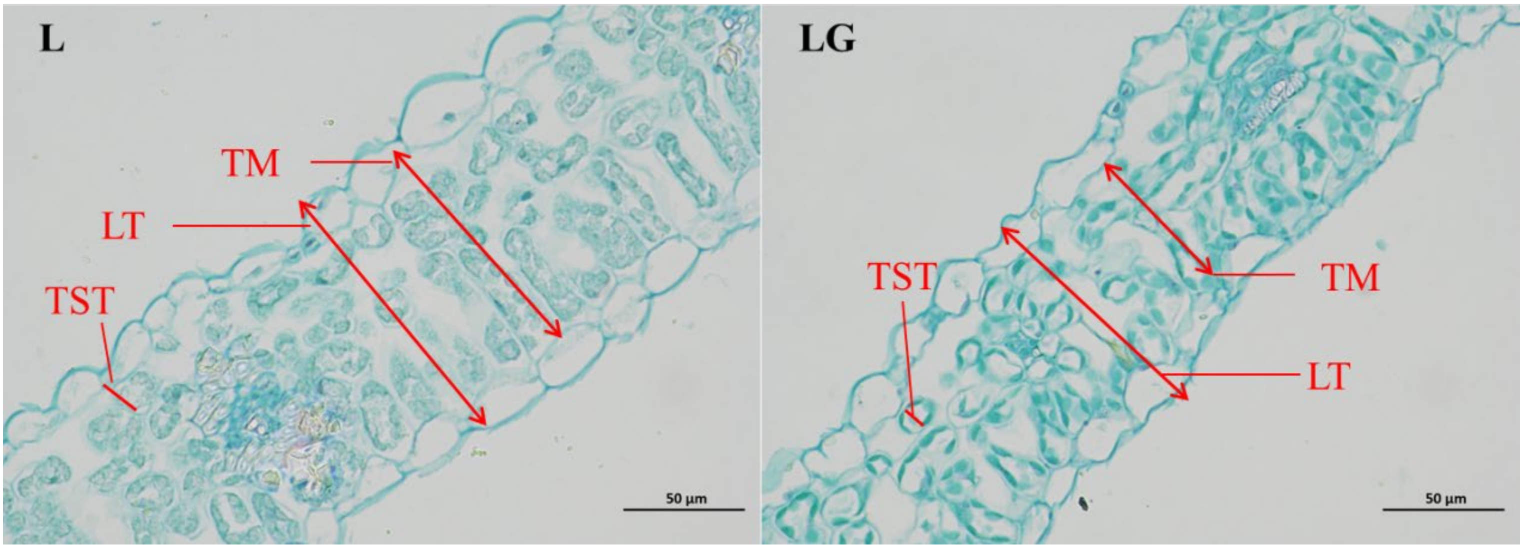


Fig. S3 Effects of GABA spraying on the anatomical structure of leaves at low temperature on day 35. L indicates the anatomical structures of leaves sprayed with distilled water; LG indicates the anatomical structures of leaves sprayed with GABA. LT, the thickness of leaf; TM, thickness of mesophyll; TST, thickness of sponge tissue.
